# Experimental Infection Using Mouse-Adapted Influenza B Virus in a Mouse Model

**DOI:** 10.3390/v12040470

**Published:** 2020-04-21

**Authors:** Elena Prokopyeva, Olga Kurskaya, Ivan Sobolev, Mariia Solomatina, Tatyana Murashkina, Anastasia Suvorova, Alexander Alekseev, Daria Danilenko, Andrey Komissarov, Artem Fadeev, Edward Ramsay, Alexander Shestopalov, Alexander Dygai, Kirill Sharshov

**Affiliations:** 1 Department of Development and Testing of Pharmacological Agents, Federal Research Center of Fundamental and Translational Medicine, 630117 Novosibirsk, Russia; kurskaya_og@mail.ru (O.K.); sobolev_i@hotmail.com (I.S.); Mariaza@ngs.ru (M.S.); murashkinatatiana89@gmail.com (T.M.); asuvorova153@gmail.com (A.S.); al-alexok@ngs.ru (A.A.); shestopalov2@ngs.ru (A.S.); sharshov@yandex.ru (K.S.); 2 Medical Department, Novosibirsk State University, 630090 Novosibirsk, Russia; 3 Department of Etiology and Epidemiology, Smorodintsev Research Institute of Influenza, 197376 Saint Petersburg, Russia; daria.baibus@gmail.com (D.D.); andrey.komissarov@influenza.spb.ru (A.K.); afadeew@gmail.com (A.F.); warmsunnyday@mail.ru (E.R.); 4 Goldberg Research Institute of Pharmacology and Regenerative Medicine Clinic, 634009 Tomsk, Russia; ombn.ramn@mail.ru

**Keywords:** influenza B virus, mouse-adapted, amino acid substitutions, pathogenicity, influenza model, virulence, antiviral drugs, influenza vaccine

## Abstract

Every year, influenza B viruses (IBVs) contribute to annual illness, and infection can lead to serious respiratory disease among humans. More attention is needed in several areas, such as increasing virulence or pathogenicity of circulating B viruses and developing vaccines against current influenza. Since preclinical trials of anti-influenza drugs are mainly conducted in mice, we developed an appropriate infection model, using an antigenically-relevant IBV strain, for furtherance of anti-influenza drug testing and influenza vaccine protective efficacy analysis. A Victoria lineage (clade 1A) IBV was serially passaged 17 times in BALB/c mice, and adaptive amino acid substitutions were found in hemagglutinin (HA) (T214I) and neuraminidase (NA) (D432N). By electron microscopy, spherical and elliptical IBV forms were noted. Light microscopy showed that mouse-adapted IBVs caused influenza pneumonia on day 6 post inoculation. We evaluated the illness pathogenicity, viral load, and histopathological features of mouse-adapted IBVs and estimated anti-influenza drugs and vaccine efficiency in vitro and in vivo. Assessment of an investigational anti-influenza drug (oseltamivir ethoxysuccinate) and an influenza vaccine (Ultrix^®^, SPBNIIVS, Saint Petersburg, Russia) showed effectiveness against the mouse-adapted influenza B virus.

## 1. Introduction

Influenza B viruses (IBVs) belong to the family *Orthomyxoviridae* [1]. IBVs have been isolated from humans and seals (*Phoca vitulina* and *Halichoerus grypus*) [2,3]. They were first isolated in 1940, and since the 1980s two IBV genetic lineages have been identified: B/Victoria/2/87 (B/Vic) and B/Yamagata/16/88 (B/Yam). These lineages are differentiated by differences in hemagglutinin (HA) and neuraminidase (NA), with almost have no antigenic crossover in the hemagglutination inhibition assay [4,5]. It is interesting to note that, in the mid-1990s, a new reassortant IBV with Victoria-like HA and Yamagata-like NA (VicHA–YamNA) emerged which displaced the Victoria-like HA and Victoria-like NA viruses (VicHA–VicNA) and continued to circulate into the 2000s [6,7,8]. Monitoring for IBVs showed that the highest levels of relative genetic diversity of the Victoria lineage occurred during the 2010–2011 and 2016–2017 seasons [9]. Human infection by IBV can lead to serious respiratory disease, the complications of which are particularly common among children of primary school age (5–8 years) [10]. Data for the United States for each epidemic season from 200 to 2011 (excluding the 2009 pandemic) show that between 22% and 44% of all childhood influenza-related deaths were caused by IBV infection. Moreover, from 2004 to 2013, Canadian researchers found significantly higher mortality rates due to IBV compared to influenza A virus in children younger than 16 years of age [11]. In Europe, influenza B accounted for 63% of all influenza cases in the 2017–2018 epidemic season [12]. A number of studies using ex vivo (explant) cultures of human bronchus or lung showed that IBVs are capable of causing severe lower respiratory tract infections which frequently lead to fatal complications [13].

Seasonal influenza vaccines are divided into types: trivalent, which consists of influenza A/H1N1, A/H3N2, and one influenza B strain (B/Yam or B/Vic); or quadrivalent, which contains all four strains. The threat of IBV has been recently recognized [14], and seasonal influenza vaccines are moving towards quadrivalent types [15,16,17], which will have greater efficacy in the case of emergent of reassortant IBVs. Vaccines designs seek to provide protection against seasonal influenza viruses by eliciting antibody responses to surface viral HA proteins. Constant antigenic drift in HA necessitates regular updating of vaccine strains to ensure that the antigenic profile of circulating strains and vaccine components match [18]. According to the Center for Disease Control (CDC), a quadrivalent vaccine is 28% effective among the especially susceptible, namely children within the age group of 9–17 years of age [19].

Despite the fact that IBVs have repeatedly caused human epidemics, their genetic determinants of virulence and transmission are still poorly understood. Limited data on the range of hosts and the absence IBV modes of transmission complicate several areas including the study of pathogenicity factors, assessment of antiviral drugs, and vaccines effectiveness. The aim of this study is to develop non-lethal infection models, using clinically-relevant viruses, which facilitate testing of (anti-influenza) vaccine-induced protection. BALB/c mice were infected with mouse-adapted influenza B virus (B/Vic) and were characterized for illness, inflammation, viral load, and histopathology. Anti-influenza drugs and vaccine efficiency were also estimated in vitro and in vivo.

## 2. Materials and Methods

All manipulations with animals were approved by the Ethics Committee of the Federal Research Center of Fundamental and Translational Medicine (No. 2017-15).

### 2.1. Viral Adaptation

The virulence of the B virus was increased by serial passages in the lungs of 8-week-old male BALB/c (*n* = 7 per group) mice (State Research Center of Virology and Biotechnology VECTOR (FSRI SRC VB VECTOR), Novosibirsk, Russia). Seven mice were lightly anesthetized with Rometar (20 mg/kg) (Bioveta, Ivanovice na Hané, Czech Republic) and intranasally infected (i.i.) with 50 µL of phosphate-buffered saline (PBS) containing 10^4^ TCID_50/mL_ (50% tissue culture infective dose) of a wild type IBV strain B/Novosibirsk/40/2017 (*B/2017*) that was isolated from a human in 2017 in Novosibirsk, Russia and belongs to B/Victoria lineage. Three of seven mice from each passage with the most evident symptoms were sacrificed by decapitation on 3rd day post-infection (d.p.i.). The lungs of these mice were used to prepare 10% homogenates in PBS. Subsequently, new groups of mice were anesthetized with the same anesthetic and i.i. with 50 µL of 10% lung homogenate. In parallel, viral replication of viruses in the lungs of the sacrificed mice was measured by titration of a 10% homogenate using Madin-Darby Canine Kidney (MDCK) cell culture [20]. Four of seven mice from each group, at each passage, were monitored daily for 14 d.p.i for signs of illness, weight loss, or lethality. After 17 passages in total, the clinical signs were registered: significant reduction in body weight (up to 30%); hypothermia; ruffled fur; and animals huddling together. The wild type IBV strain *B/2017* and mouse-adapted variant (strain B/Novosibirsk/40/2017-MA (*B/2017-MA*)) were patented [21]. The median mouse infectious dose (MID_50_) of the virus *B/2017-MA* was 4.6 ± 0.26 log_10_/mL, or 1.88 TCID_50_; the TCID_50_ of *B/2017-MA* was 4.9 ± 0.21 log_10_/mL. Both strains (wild type strain and *B/2017-MA)* are non-lethal for mice.

To evaluate the pathogenicity of the *B/2017* and *B/2017-MA* viruses, groups of six 6-week-old male BALB/c mice (*n* = 10 per group) were anesthetized with Rometar (20 mg/kg) and i.i. with 50 μL of PBS containing 10^4^ TCID_50_/mL and 10 MID_50_, respectively. Intact mice (*n* = 3 per group) were i.i. with 50 µL of PBS (pH 7.2) and served as the control. Body weight and temperatures changes, as well as mouse survival rate were monitored daily for 14 d.p.i. Body weight was measured by using a laboratory animal weighing analytical balances MASSA-K VK-1500 (MASSA-K, Saint Petersburg, Russia), and body surface temperature was taken from the ear canal using a hand-held infrared thermometer ‘AccuVET’ (Mesure Technology Co., Ltd., West Bromwich, UK).

To detect the tissue distribution of *B/2017* and *B/2017-MA* viruses, on days 3 and 6 p.i., three mice were sacrificed, and organ samples of lungs, brain, heart, liver, kidneys, and spleen were harvested in 1 mL of PBS. Samples were then homogenized and centrifuged, and viral titers in the homogenized supernatants were determined by the Kerber method with Ashmarin–Vorobyov modification. To assess by light and electron microscopy pathological lesions in mice infected with *B/2017* or *B/2017-MA* viruses, their lungs were harvested at the 3rd and 6th d.p.i.

### 2.2. Light Microscopic Examination

Lungs from 3 animals in each group (B/2017 infected and B/2017-MA infected) were examined by light microscopy on the 3rd and 6th d.p.i. and subsequently fixed in 4% formalin solution, dehydrated (according to the standard procedure), and embedded into paraffin. Then, 4–5 microns-thick paraffin sections were obtained using an HM 340 E rotary microtome (Carl Zeiss, Jena, Germany) and stained by the hematoxylin and eosin (H&E) method. Light microscopy and photography were carried out using an Axioskop 40 microscope (Carl Zeiss, Jena, Germany).

### 2.3. Electron Microscopic Examination

Lung samples were taken on the 3rd and 6th d.p.i. with *B/2017* and *B/2017-MA* viruses. Samples were: fixed with 2.5% glutaraldehyde in 0.1 M phosphate buffer pH 7.4 for 4 h at 4 °C; re-fixed with 1% osmium tetroxide in 0.1 M phosphate buffer pH 7.4 at 4 °C for 2 h; then dehydrated in ethanol (50°, 70°, 96°, 100°) followed by acetone and Araldite-Epon mixture (1:6) (SPI, West Chester, PA, USA) with the addition of the catalyst 2,4,6-tris(dimethylaminoethyl)-phenol (DMP-30) and polymerized at 60 °C. Semi-thin sections were prepared from solid blocks, stained with Azur II and examined in a light microscope to highlight areas for ultrathin sectioning. Ultrathin sections were cut with an EM UC7 ultramicrotome (Leica, Wien, Austria). Sections were stained with uranyl acetate, followed by lead citrate (SPI, West Chester, PA, USA). The samples were examined on a transmission electron microscope LIBRA 120 (Carl Zeiss, Jena, Germany) at 100 kV, and images were captured using a Veleta digital camera (EMSIS GmbH, Muenster, Germany).

### 2.4. Sequencing and GISAID Accession Numbers

Viral RNA was extracted using the QIAamp Viral RNA Mini Kit (QIAGEN, Germantown, MD, USA) according to the manufacturer’s instructions. Whole genome amplification of the influenza B genome was performed using the SuperScriptTM III One-Step RT-PCR System with Platinum^TM^ Taq High Fidelity DNA Polymerase (Thermo Fisher Scientific, Waltham, MA, USA) with modifications [22].

Products of PCR were analyzed by agarose gel electrophoresis, and sequencing was performed using the Illumina MiSeq platform. Paired-end libraries for the MiSeq platform were prepared using the Nextera XT DNA Library Prep Kit (Illumina, San Diego, CA, USA). The sequencing library was quantified using the NEBNext Library Quant Kit (NEB, Evry, France). Library size was assessed using an Agilent Bioanalyzer 2100.

The MiSeq v2 reagent kit (300-cycle; 2 × 150-bp PE) (Illumina, San Diego, CA, USA) was used for sequencing. Nucleotide sequences of the *B/2017* and *B/2017-MA* strains are available in the GISAID database with the following accession numbers: EPI_ISL_338315 and EPI_ISL_338316, respectively.

### 2.5. Genetic Analysis

The IBV nucleotide sequences being investigated were combined with sequences retrieved from the GISAID database. For multiple alignments, a MUSCLE program was used [23]. Comparative pairwise sequence alignment of 2 investigated strains was performed via BioEdit. Phylogenetic trees were built via MEGA 5 using maximum likelihood and utilizing the general time reversible (GTR) nucleotide substitution model. Bootstrap support values were generated using 500 rapid bootstrap replicates.

### 2.6. Determination of Susceptibility to Neuraminidase Inhibitors

The susceptibility of the *B/2017* and *B/2017-MA* strains to oseltamivir (Hoffmann-La Roche, Basel, Switzerland) was evaluated by published NA inhibition assays [24,25]. Briefly, viruses were standardized to a NA activity level 10-fold higher than that of the background, as measured by the production of fluorescent product from methylumbelliferyl-N-acetylneuraminic acid (MUNANA) substrate (Sigma-Aldrich, Darmstadt, Germany). Drug susceptibility profiles were determined by the extent of NA inhibition (NAI) after incubation with 3-fold serial dilutions of NAIs. The 50% inhibitory concentrations (IC_50_) were determined from the dose-response curve.

**Figure 1:**
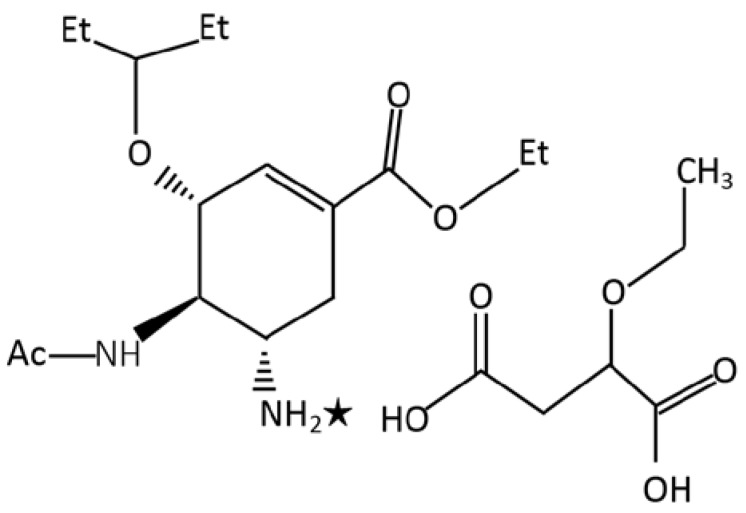
Ethyl (3S,4R,5S)-4-acetamido-5-amino-3-(1-ethylpropoxy)cyclohex-1-en-1-carboxylate ethoxy succinate formula. ✭ -hydrogen bonding.

In this study, a new neuraminidase inhibitor (ethyl (3S,4R,5S)-4-acetamido-5-amino-3-(1-ethylpropoxy)cyclohex-1-en-1-carboxylate ethoxy succinate (oseltamivir ethoxysuccinate) [26] (Figure 1) which features antiviral activity was used. The novel compound is prepared by treatment of ethyl(3S,4R,5S)-4-acetamido-5-amino-3-(1-ethylpropoxy)cyclohex-1-en-1-carboxylate with ethoxy succinic acid in ethyl acetate.

### 2.7. Determination of Anti-Influenza Drugs Efficacy

We studied anti-influenza efficacy of oseltamivir ethoxysuccinate and Tamiflu^®^ on 6–8-week-old BALB/c mice (FSRI SRC VB ‘’VECTOR”, Koltsovo, Russia) (*n* = 10 per group). All mice of groups №1–3 were lightly anesthetized with Rometar (2 mg/kg) and then i.i. with 10 MID_50_ of *B/2017-MA* strains in 50 µL. Mice of group №4 were i.i. with 50 µL of PBS and served as the control. Then mice of group №1 were treated per os (oral administration) with a dose of 25 mg/kg/day of oseltamivir ethoxysuccinate (200 µL in each) during the 5 d.p.i. Mice in group №2 were treated per os with the same dose of Tamiflu^®^ during the 5 d.p.i. Mice from group №3 received 200 μL of distilled water per os during the 5 d.p.i. All animals were monitored for signs of illness, weight loss, temperature changes, mortality, and lethality over the 14 d.p.i.

### 2.8. Mouse Immunization and Inoculation

The, 6–8-week-old male BALB/c mice (FSRI SRC VB “VECTOR”, Koltsovo, Russia) were randomly distributed into 3 groups (*n* = 10 per group). Mice were twice (prime boost) immunized subcutaneously (i.s.) with 0.25 mL Ultrix^®^ vaccine (SPBNIIVS, Saint Petersburg, Russia) containing purified surface antigens from the influenza strain B/Colorado/06/2017 (lineage B/Victoria/2/87).

On the 14th day after the second immunization, mice were lightly anesthetized with Rometar (20 mg/kg) (Bioveta, Ivanovice na Hané, Czech Republic) and i.i. with 50 µL of PBS containing 10^4^ TCID_50_ of the *B/2017-MA* virus or sterile PBS. On the 3rd and 6th d.p.i., 3 animals from each group were humanely euthanized for tissue collection. Lungs were collected for virus titer quantification and examination by light and electron microscopy. On the 21st d.p.i., mice were bled from the submandibular vein for serology. Clinical signs of illness, such as body weight and temperature changes, mortality, and morbidity were monitored daily throughout the study.

### 2.9. Statistical Analyses

All in vitro assays were performed at least twice in triplicate. Virus titers were calculated by the Kerber method with Ashmarin–Vorobyov modification [27], as follows: log_10_TCID_50_/mL = lgDn − δ(ΣLi − 0.5).

For multiple comparisons, two-way analysis of variance (ANOVA) was performed. A *p*-value below 0.05 was considered significant.

## 3. Results

### 3.1. Viral Adaptation

To study the adaptation of B virus isolated from humans, we serially passaged the wild type IBV Victoria lineage strain *B/2017* in BALB/c mice. After 17 passages total, the mouse-adapted B virus *B/2017-MA* was obtained. We compared the pathogenicity of the *B/2017* and *B/2017-MA* strains. Groups of twelve 6–8-week-old male BALB/c mice were i.i. with 50 μL of B/2017 or *B/2017-MA* viruses at 10^4^ TCID_50_ (10 MID_50_). Body weight, temperature changes, morbidity, and mortality were monitored during 14 d.p.i, and the PBS-inoculated group of mice served as the controls. On the 3rd and 6th d.p.i., 3 animals from each group were sacrificed by decapitation, and internal organ samples (lungs, brain, heart, liver, kidneys, spleen) were taken for comparative virological analysis. On day 3 and 6 p.i., lungs were taken for examination by electron microscopy.

**Figure 2:**
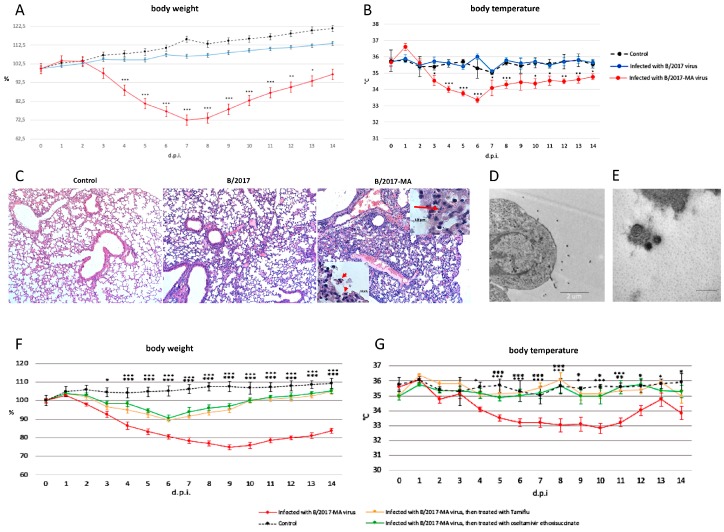
Comparative analysis of *B/2017* and *B/2017-MA* strains, and assessment of anti-influenza drugs against mouse-adapted influenza B virus in vivo. Note: (**A**) body weight and (**B**) body temperature; intact mice received phosphate-buffered saline (PBS) (dotted black line); mice intranasally infected with 10^4^ TCID_50_ (50% tissue culture infective dose) of *B/2017* strain (blue line); mice intranasally infected with 10 MID_50_ (mouse infectious dose) of *B/2017-MA* strain (red line). ✶ *p* < 0.05; ✶✶ *p* < 0.01; ✶✶✶ *p* < 0.001, reliability when comparing data from groups of infected mice. (**C**) Lungs from mice euthanized 6 days post inoculation were collected and were preserved in 10% formalin for histopathological examination by hematoxylin and eosin (H&E) staining. Images were taken at × 40 magnification. Insertions: arrows indicate the apoptotic cells. Images were taken at ×1000 magnification. (**D**) Budding of influenza B virions from the surface of type 1 alveolar cells on the 3rd day post infection (d.p.i.) with *B/2017-MA* strain. bar = 20 microns. (**E**) Virions of influenza B; bar = 200 nm. (**F**) Body weight and (**G**) temperature of: intact mice who received PBS (dotted black line); mice infected intranasally with 10 MID_50_ of *B/2017-MA* strain (red line); mice infected with 10 MID_50_ of *B/2017-MA* strain and treated with Tamiflu^®^ (orange line); mice infected with 10 MID_50_ of B/2017-MA strain and treated with oseltamivir ethoxysuccinate (green line).✶ *p* < 0.05; ✶✶ *p* < 0.01; ✶✶✶ *p* < 0.001, reliability when comparing data from groups of infected mice.

All mice i.i. with *B/2017* virus survived and showed no obvious clinical signs such as body weight or temperature changes (Figure 2A,B). In contrast, another experimental group of mice i.i. with *B/2017-MA* showed gradually weight loss: approximately 10% at 3 d.p.i.; 15% at 4 d.p.i.; 20% at 5 d.p.i.; 25% at 6 d.p.i.; and 30% at 7 d.p.i. (Figure 2A). Temperature measurements indicated the peak infection time frame to be from the 4th to the 7th d.p.i. (Figure 2B).

At 3 and 6 d.p.i., three mice were sacrificed, and their organs (lungs, brain, heart, liver, kidneys, spleen) were harvested. Viral titers in the collected organs were determined by the Kerber method with Ashmarin–Vorobyov modification. The strain *B/2017* could replicate in mouse lungs only 3 days with titers 2.9 ± 0.2 log_10_TCID_50_/mL. In contrast, strain *B/2017-MA* replicated very well in mouse lungs, with higher titers and a longer period: on the 3rd d.p.i., the titer was the 5 ± 0.1 log_10_TCID_50_/mL; and on the 6th d.p.i. it was 3.3 ± 0.3 log_10_TCID_50_/mL. Therefore, the mouse-adapted B virus strain *B/2017-MA* displayed much higher replication capability in mouse lungs. In other organs (such as brain, heart, liver, kidneys, or spleen) we did not find a viral load from either strain.

Histopathological analysis of mouse lungs infected with the *B/2017* virus showed slight damage, such as: small numbers of eosinophilic cells in the bronchioles; low blood filling of the capillaries; and edema with a high protein content on the 6th d.p.i. (Figure 2C). In contrast, on the same d.p.i., pathomorphological changes in lungs of mice infected with the *B/2017-MA* virus were more pronounced due to viral triggered apoptosis, leading to desquamation of the bronchial epithelium (Figure 2C, insertion). Other features noted included: a greater number of eosinophilic cells in the bronchioles; lymphocytic infiltration of various lung regions; and capillaries stasis (Figure 2C). All of the pathomorphological changes listed were predominantly located in the root, cranioventral, and middle regions of the left and right lungs of the *B/2017-MA* virus infected mice.

Electron microscopic examination revealed budding of virions from the surface of type 1 alveolar cells on the 3rd d.p.i. in samples from mice in the *B/2017-MA* group (Figure 2D,E). It is interesting to note that various virion morphologies were seen, such as spherical or elliptical, but not filamentous.

### 3.2. Sequencing and Genetic Analysis

According to nucleotide sequence analysis of all eight genome segments, the *B/2017* virus and, consequently, its mouse-adapted variant *B/2017-MA* belong to the B/Vic genetic lineage. In addition, analysis of these strains of HA amino acid sequences revealed mutations (I117D, N129D, V146I) relative to earlier reference strains. These mutations are characteristic of strains belonging to the 1A genetic subgroup of the B/Vic lineage. The strains NA substitutions were also found to feature amino acid substitutions characteristic of genetic group 1A of the B/Vic lineage (N340D, E358K, S295R, I120V, and K220N). To assess the phylogenetic relationships between *B/2017* and *B/2017-MA*, all genome segments were analyzed using phylogenetic dendrograms. Analysis used IBV nucleotide sequences, available in the GISAID database, isolated from residents of Russia and Kazakhstan, as well as vaccine and reference strains according to the World Health Organization (WHO) classification.

According to dendrograms (Supplementary Materials, Figure S1–S8), the studied strains form a common phylogenetic group with other isolates from Novosibirsk, as well as strains from the neighboring Altai Republic and the Republic of Kazakhstan. At the same time, all of them are phylogenetically distanced (although only slightly) from IBV strains isolated in other Russian regions.

To identify strains that are the most genetically related to *B/2017* and *B/2017-MA*, BLAST analysis was performed. The results revealed that the study strains are 99–100% identical to the IBV variants that circulated in the human population in the Novosibirsk region, Altai Republic, and the Republic of Kazakhstan (Table S1). Thus, strain *B/2017* is a typical genetic variant of the IBV that circulated during the 2016–2017 epidemic season, and it is most genetically related to the strains that circulated in Asia at that time.

Comparative analysis of nucleotide and amino acid substitutions between the two strains (*B/2017* vs. *B/2017-MA*) showed the presence of synonymous (not leading to amino acid substitution) nucleotide substitution in the PB1 segment—A2175G. Additionally, nucleotide substitution in the HA segment (C641T) led to amino acid substitution in the HA protein (T214I). According to the FluSurver resource [28], the detected substitution is rare and present in 0.46% HA of IBVs isolated from 2008 to 2016. The identified amino acid substitution is localized in the antigenically active subunit HA-HA1, which can potentially affect the biological properties of the virus. In the sequence coding NA, a nucleotide substitution (G1294A), which leads to the amino acid change D432N, was detected. According to the FluSurver resource [28], this substitution has only been found in one strain (B/Hawaii/37/2017) so far.

### 3.3. Assessment of Antiviral Drug Therapy In Vitro and In Vivo

Here, we measured the IC_50_ of oseltamivir ethoxysuccinate that is necessary to reduce the NA activity of the *B/2017* and *B/2017-MA* strains (Table 1). Thus, analysis of the NA inhibition in vitro showed that oseltamivir ethoxysuccinate and Tamiflu^®^ reduced the neuraminidase activity of the *B/2017* and *B/2017-MA* strains equally effectively.

The study also analyzed anti-influenza drug efficacy in vivo. No significant body weight or temperature changes between groups of animals i.i. with the *B/2017-MA* strain, and then treated per os for 5 days with oseltamivir ethoxysuccinate or Tamiflu^®^, were seen (Figure 2F,G). All mice treated with anti-influenza drugs lost no more than 10% of body weight and began to recover on the 7th d.p.i. In addition, no hypothermia was detected among them. Therefore, it was shown that the innovative drug (oseltamivir ethoxysuccinate) displays high effectiveness, like Tamiflu^®^, against the mouse-adapted B virus.

### 3.4. Assessment of Vaccine Efficiency Against Mouse-Adapted Influenza B Virus In Vivo

We assessed vaccine efficiency against the mouse-adapted *B/2017-MA* strain in vivo. In preparation, BALB/c mice were immunized with purified surface antigens from the influenza strain B/Colorado/06/2017 (lineage B/Victoria/2/87) (Ultrix^®^, SPBNIIVS, Saint Petersburg, Russia) in 0.25 µL twice (prime boost). On day 21 after vaccination, mice were i.i. with the *B/2017-MA* strain. Infected but not immunized mice served as controls.

**Figure 3:**
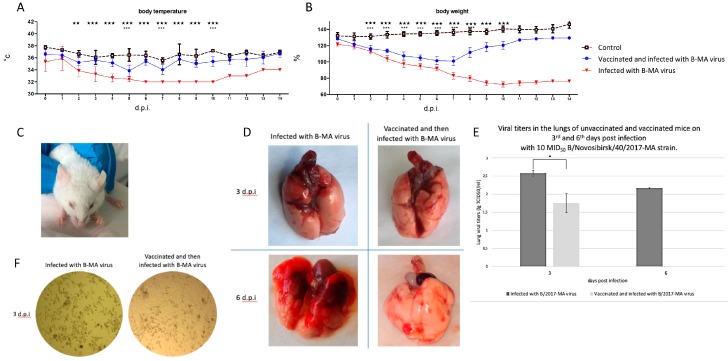
Assessment of vaccine efficiency against mouse-adapted influenza B virus in vivo and in vitro. Note: (**A**) body temperature and (**B**) body weight of; intact mice received PBS (dotted black line); vaccinated and infected intranasally with 10 MID_50_ of *B/2017-MA* strain mice (dark blue line); infected intranasally with 10 MID_50_ of *B/2017-MA* strain mice (red line); ✶ *p* < 0.05; ✶✶ *p* < 0.01; ✶✶✶ *p* < 0.001, reliability when comparing data from groups of infected mice. (**C**) On day 3 day, conjunctivitis in a mouse infected with 10 MID_50_ of *B/2017-MA* strain. (**D**) Lungs of unvaccinated and vaccinated mice on 3rd and 6th days post infection with 10 MID_50_ of *B/2017-MA* strain. (**E**) Viral titers in the lungs of unvaccinated and vaccinated mice on 3rd and 6th days post infection with 10 MID_50_ of *B/2017-MA* strain. (**F**) Cytopathic effect of Madin-Darby Canine Kidney (MDCK) cell culture on 3rd day post infection of unvaccinated and vaccinated mice with 10 MID_50_ of *B/2017-MA* strain.

Infection induced by *B/2017-MA* in non-immunized mice was characterized by body weight loss and hypothermia from the 2nd d.p.i, as well as conjunctivitis (up to 30%), with onsets between 1 and 3 d.p.i. (Figure 3A–C). All mice in this group huddled together, and their fur was ruffled on the 4th d.p.i. Peak illness was determined to be from the 5th to the 10th d.p.i., as seen by a large percentage of total body weight lost and visibly increased breathing effort caused by severe pathological processes in the lungs (Figure 3D). All mice unimmunized and i.i. with *B/2017-MA* lost significant amounts of body weight relative to the comparison groups (*p* < 0.001). In addition, mice in the vaccinated and *B/2017-MA* infected group experienced statistically-significant reductions in body weight from the 4th up to the 7th d.p.i., compared to the control group (*p* < 0.001). Temperatures were also measured, and statistically-significant differences (*p* < 0.001) between the vaccinated and *B/2017-MA* infected mice and intact mice were detected on the 5th, 7th, and 10th d.p.i. The data suggest that the adapted IBV affects vaccinated mice for up to 10 d.p.i. No other influenza signs were observed in vaccinated mice, which indicate a mild IBV illness and effective Ultrix^®^ vaccination.

Lung viral loads were assessed via MDCK cell culture (all groups) (Figure 3E,F). The virus was detected in lungs via TCID_50_ assay on day 3 for all infected mice (Figure 3F). On day 6, the virus was only detected in unvaccinated mice (Figure 3F). In the vaccinated and *B/2017-MA* infected group, it was noted that only 10–20% of cells in the walls were affected, but a 100% cytopathic effect was detected in the comparison group (Figure 3F).

Macroscopic examination of unvaccinated mouse lungs revealed inflammation in the root and cranioventral regions on the 3rd d.p.i. In contrast, minor patchy areas of alveolar hemorrhage (Figure 3D) were seen in the vaccinated and *B/2017-MA* infected group. By the 6th d.p.i., the inflammatory process had worsened only in lungs of unvaccinated mice, as characterized by interstitial pneumonia, affecting almost the entire lung, except for the caudal sections (Figure 3D). There were no significant differences in lung inflammation on day 6 after challenge in vaccinated mice (Figure 3D).

## 4. Discussion

Influenza B viruses have real epidemiological significance, especially among children. Together, IBV and influenza A cause significant seasonal burdens. A lack of information on the host range of IBVs and the need for an adequate infection model have complicated several areas including: the study of pathogenicity factors and transmission methods; the ability to evaluate the antiviral drugs effect; and assessment of vaccine effectiveness. The main drawback to the mouse model is the need to use mouse-adapted viruses. The most famous mouse-adapted virus (strain B/Lee/1940) is currently considered as antiquated because it is not genetically and antigenically representative of influenza viruses currently circulating among humans.

In the field of virology, there is an entire branch devoted to obtaining recombinant strains for vaccines. IBV strains that are commonly used in experiments in Russian laboratories are recombinant strains obtained for vaccines [29,30,31,32,33,34,35]. Such IBV strains, however, are attenuated and apatogenous for experimental animals, which, in turn, does not provide an opportunity to study the pathological process of influenza infection and makes it difficult to perform studies of anti-influenza drug effectiveness in vivo. Consequently, they do not provide an opportunity to study the pathological process of influenza infection. Moreover, many of the presented recombinant strains have lost their antigenic relevance today.

Since preclinical trials of anti-influenza drugs are conducted mainly in mice [36], we also sought a mouse model. An antigenically-relevant B/Vic virus (strain *B/2017* (VicHA–VicNA), which had circulated and predominated in 2017) was chosen and adapted in mice in order to receive the antigenically- and clinically-relevant strain (*B/2017-MA*) for use in in vitro studies, and also in experimental infection on mice to evaluate the therapeutic and preventive effectiveness of antiviral drugs and vaccines in vivo. In our work we demonstrate productive infection and clinically apparent signs of disease of mouse-adapted IBV Victoria lineage which was first obtained in Russia. Both strains (*B/2017* and *B/2017-MA*) were deposited in the State collection of causative agents of viral infections and rickettsioses of the State Research Center of Virology and Biotechnology “Vector” (Koltsovo, Russia) under registration numbers V-810 and V-811, accordingly. Amino acid substitutions associated with IBV adaptation to mice were identified. They likely increase the pathogenicity and enhanced replication ability in infected mammals.

On the 6th d.p.i in BALB/c mice, influenza pneumonia featuring leukocyte and lymphocyte infiltration of bronchioles was detected in the lungs of animals infected with mouse-adapted IBV (strain *B/2017-MA*). On the same day, no viral loads were detected in the lungs of mice infected with wild type IBV (strain *B/2017*). Despite this fact, pathomorphological changes were registered and characterized as mild disease. Since the knowledge of the IBV virion structure remains limited [37,38], we utilized electron microscopy. Elliptical forms were noted in addition to spherical ones. This may possibly indicate that influenza B virions are pleomorphic in structure.

Comparative analysis of nucleotide and amino acid substitutions between wild strain *B/2017* and its mouse-adapted variant *B/2017-MA* strain revealed non-synonymous nucleotide substitutions that led to amino acid substitutions in two proteins, HA and NA. One of the amino acid substitutions identified (T214I in HA) is localized in the antigenically active subunit HA-HA1, thereby affecting the biological properties of the virus. Interestingly, the same substitution was present in 0.46% of IBVs reported from 2008 to 2016 [28]. This fact may explain severe cases and poor clinical outcomes in some patients infected with IBVs. Another substitution was found in NA and characterized as rare [28]. In light of the above, we assume that the Asn amino acid at position 432 of the NA protein in complex with other substitution(s) in the surface glycoprotein HA might jointly be responsible for the high pathogenicity.

Due to their roles in elevating seasonal morbidity and mortality among humans worldwide, influenza A and B viruses have epidemiological, social, and economic significance [4,17]. Selective inhibition of neuraminidase is used in the treatment of influenza to control the processes of budding and release of mature virions from the surface of an infected host cell (as a result of cleavage of sialic acid residues from hemagglutinin). In addition, NA plays a key role in the initial stages of infection, ensuring the penetration of influenza viruses into cells. Due to the specific activity of NA, inhibitors work effectively against influenza A and B viruses. Oseltamivir, zanamivir, peramivir, and laninamivir are long-acting neuraminidase inhibitors for the treatment and prophylaxis of human influenza virus infection [39,40]. Between the NA inhibitors of oseltamivir phosphate (Tamiflu^®^) and zanamivir (Relenza^®^), the former is considered the most effective because of its higher bioavailability (30–100%) [25]. However, the use of anti-influenza drugs to prevent and treat diseases is complicated by viral resistance which has been observed in recent years [4,41]. In addition, it has been shown that Tamiflu^®^ is a lot less effective in treating influenza B, compared to influenza A [42]. Due to the facts that the investigational drug (oseltamivir ethoxysuccinate) is a modified version of Tamiflu^®^ and that it has shown (like Tamiflu^®^) high effectiveness against the mouse-adapted IBV, oseltamivir ethoxysuccinate is a promising drug for the treatment of influenza caused by the B virus.

More attention should be paid to mammalian adaptation of IBVs and how such procedures and mechanisms can enhance the development of vaccines against current influenza strains. Due to the fact that no significant lung damage was detected in vaccinated mice on day 6 after challenge, we can conclude that the Ultrix^®^ quadrivalent vaccine is effective against our mouse-adapted IBV.

## 5. Conclusions

In this work, we serially passaged a Victoria lineage IBV 17 times in BALB/c mice. The mouse-adapted IBV caused influenza pneumonia on day 6 post inoculation. Apparently, selective accumulation of amino acid substitutions in the mouse-adapted IBV, including changes to HA (T214I) and NA (D432N), may increase pathogenicity following the adaptation and be important for the virions to attach to hosts airways. However, the specific effects of these amino acid substitutions on mammalian pathogenicity requires further study. Spherical and elliptical IBV virion morphologies were also shown. Assessment of the investigational anti-influenza drug oseltamivir ethoxysuccinate and influenza vaccine Ultrix^®^ showed effectiveness against our mouse-adapted influenza B virus. In summary, we demonstrated productive infection and clinically apparent signs of disease of Victoria lineage mouse-adapted IBVs (strain *B/2017-MA*) which are useful for estimation of anti-influenza drugs and protective efficacy of influenza vaccines in vitro and in vivo.

## Figures and Tables

**Table 1:** Assessment of influenza B viruses in neuraminidase (NA) enzyme inhibition assays.

Strains	Oseltamivir Ethoxysuccinate IC_50_ (nM)	Oseltamivir Carboxylate IC_50_ (nM)
*B/2017*	75.7 ± 10.4	107.7 ± 54.0
*B/2017-MA*	122.7 ± 24.0	43.5 ± 8.1

## Supplementary Materials

The following are available online at https://www.mdpi.com/1999-4915/12/4/470/s1.
