# Supplementary material for: Experimental Infection Using Mouse-Adapted Influenza B Virus in a Mouse Model
Source: Viruses. 2020 Apr 21;12(4):470. doi: 10.3390/v12040470 (PMC7232149; doi:10.3390/v12040470)
Supplement: Supplementary file 1 [file viruses-12-00470-s001.pdf]

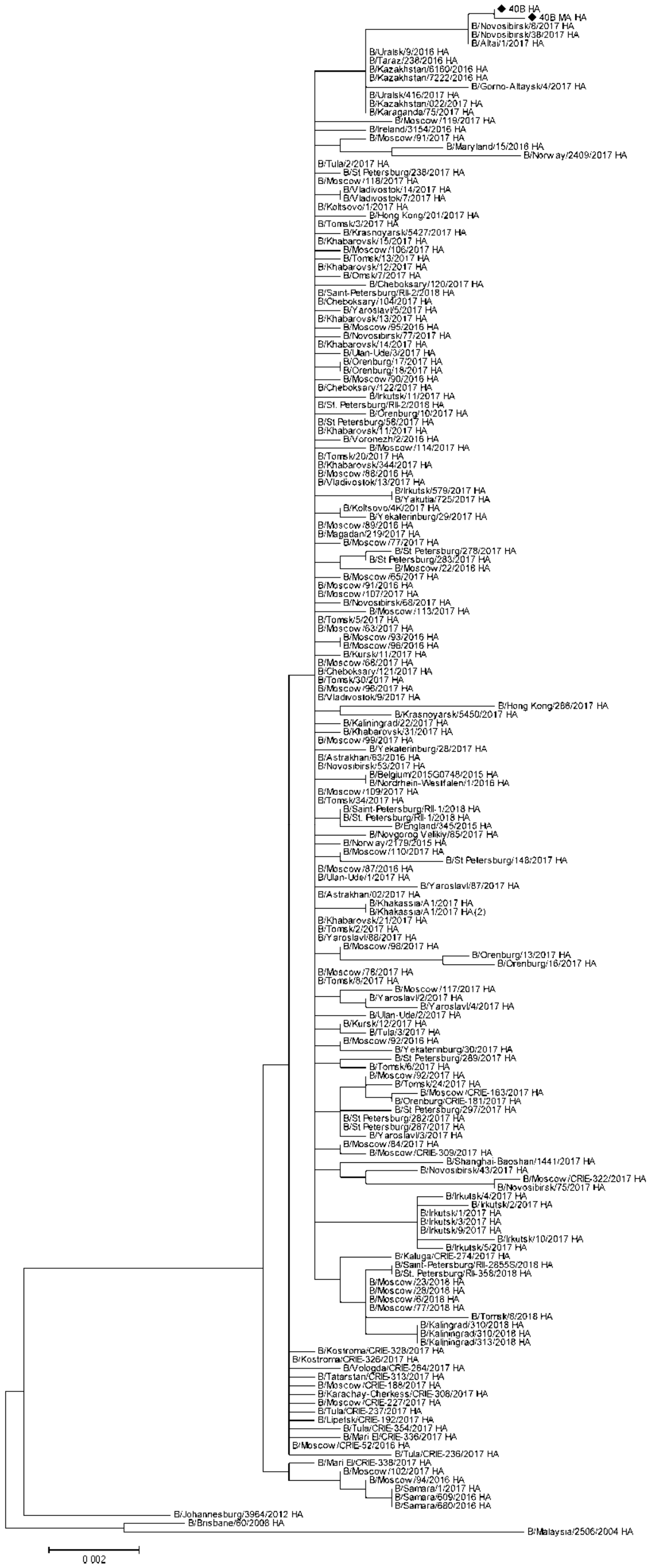

Figure 1. Phylogenetic dendrogram of a genome segment encoding HA of the influenza B virus. Studied strains are marked with black rhombuses.

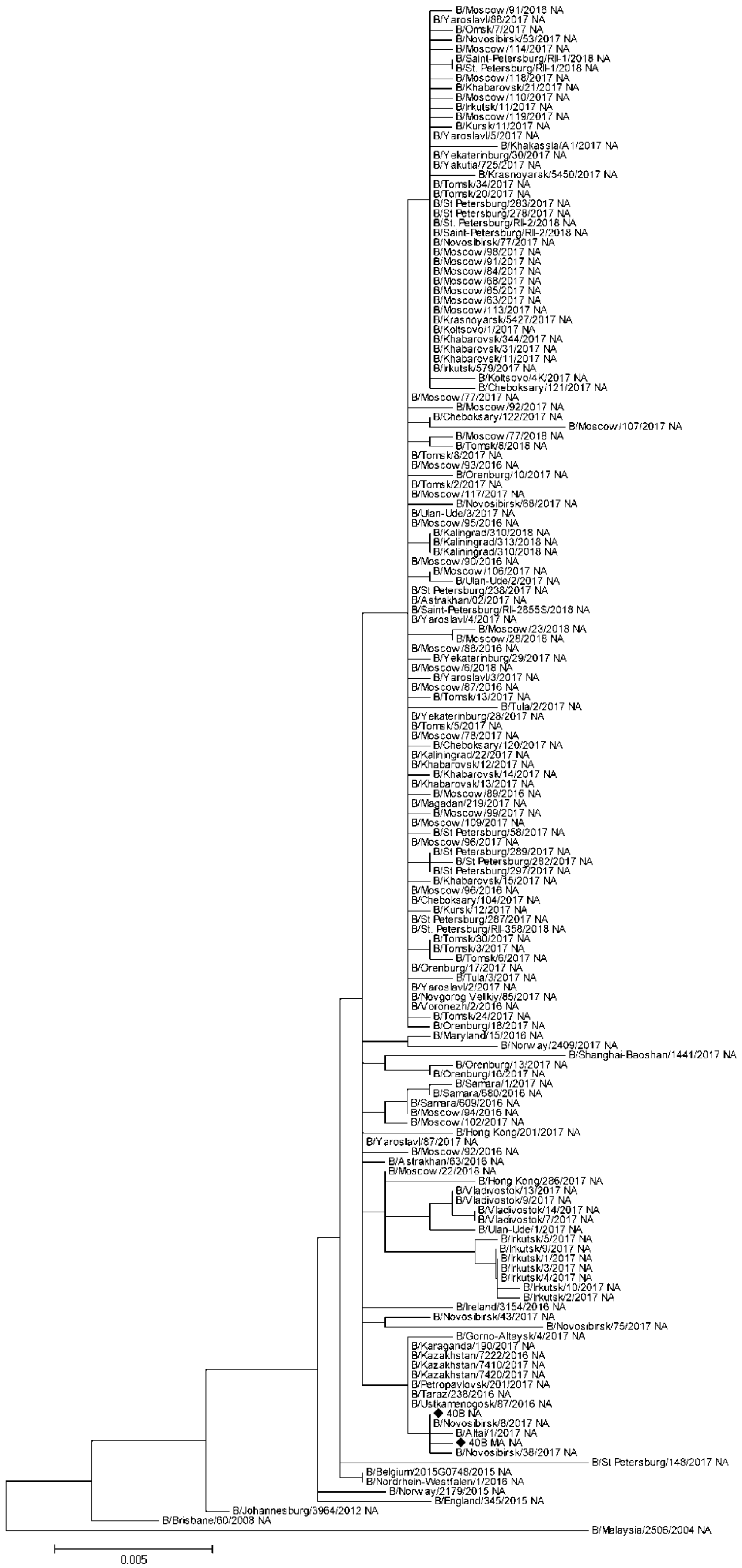

Figure 2. Phylogenetic dendrogram of a genome segment encoding NA of influenza B virus. Studied strains are marked with black diamonds.

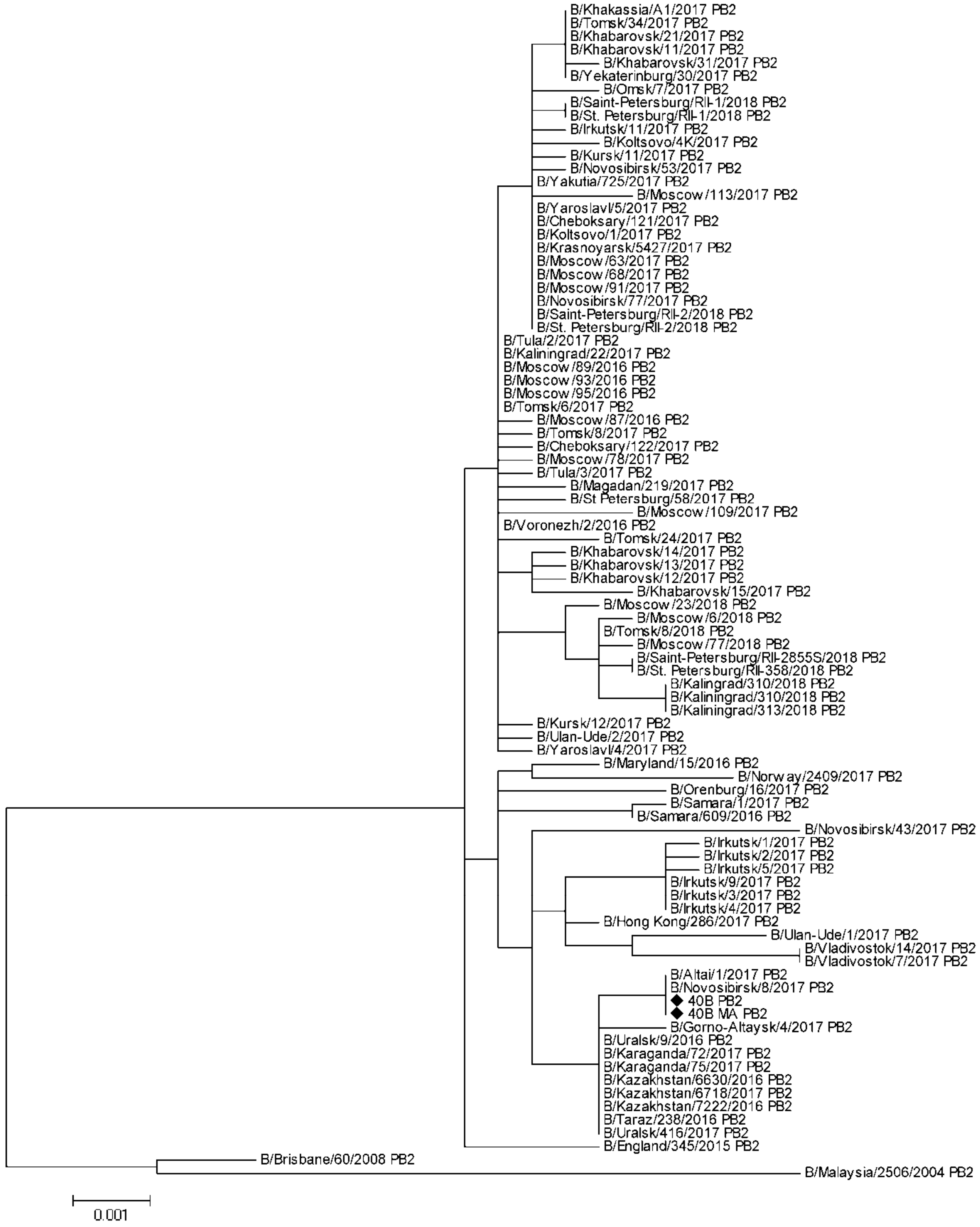

Figure 3. Phylogenetic dendrogram of a genome segment encoding PB2 of influenza B virus. Studied strains are marked with black diamonds.

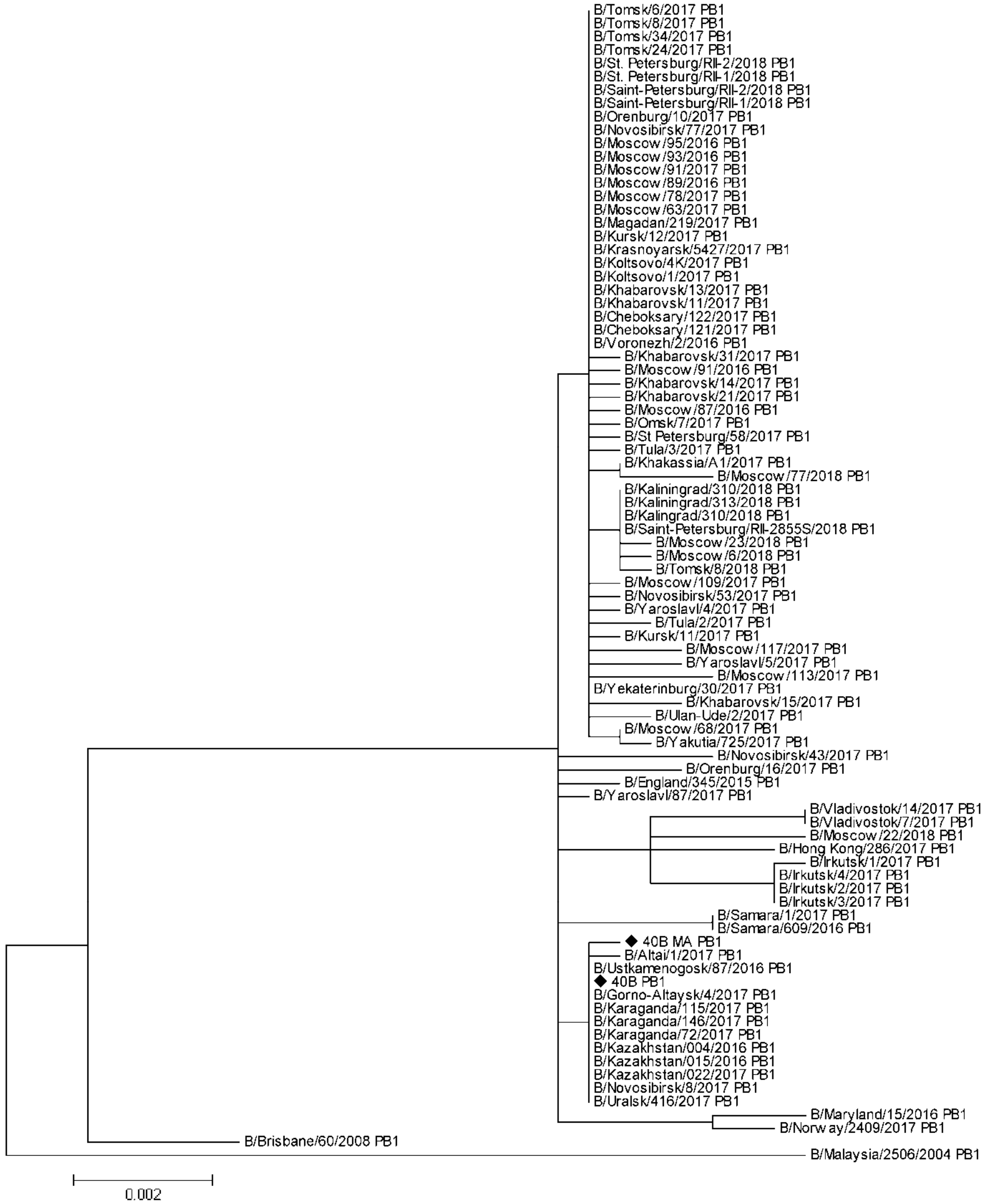

Figure 4. Phylogenetic dendrogram of a genome segment encoding PB1 of influenza B virus. Studied strains are marked with black diamonds.

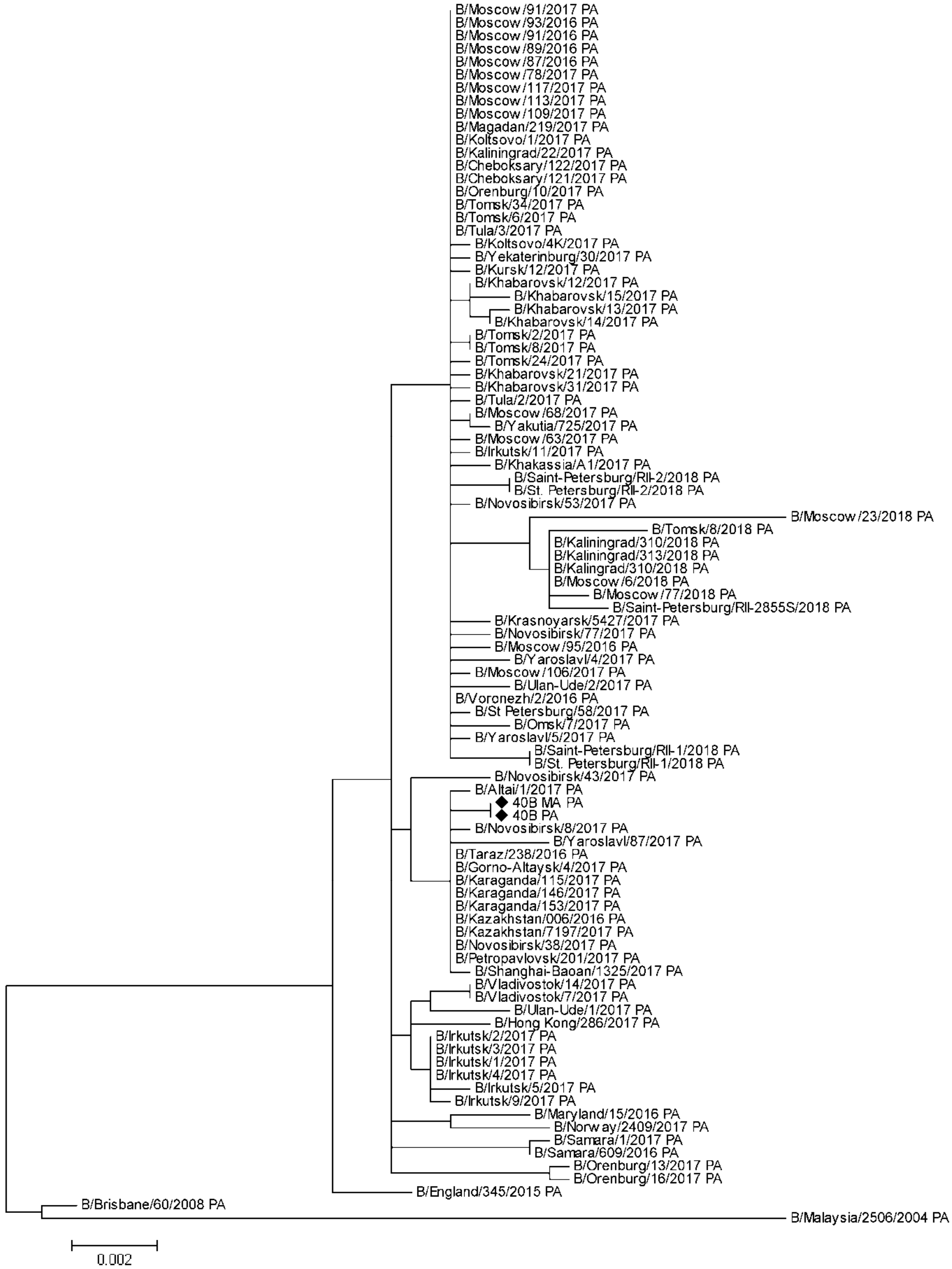

Figure 5. Phylogenetic dendrogram of a genome segment encoding PA of influenza B virus. Studied strains are marked with black diamonds.

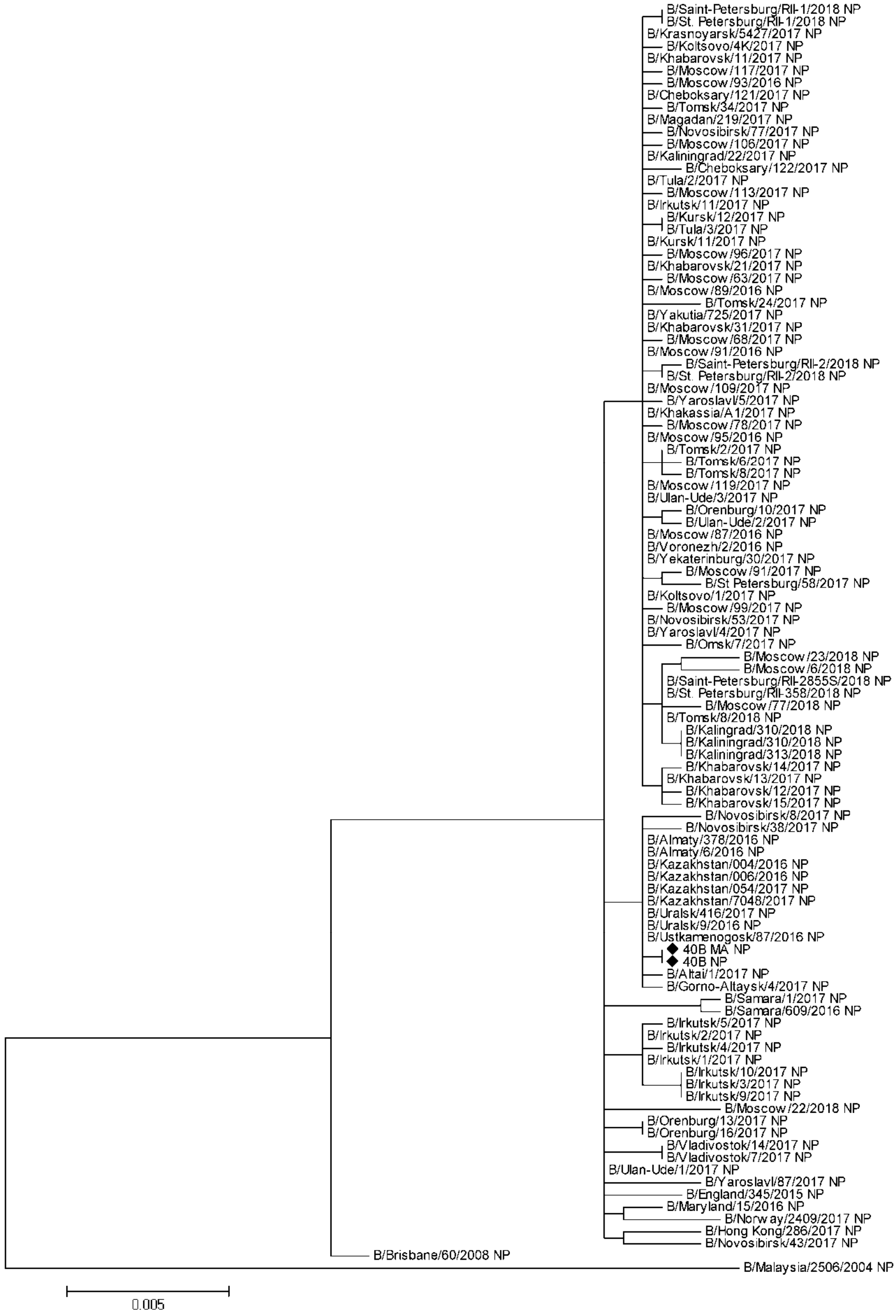

Figure 6. Phylogenetic dendrogram of a genome segment encoding NP of influenza B virus. Studied strains are marked with black diamonds.

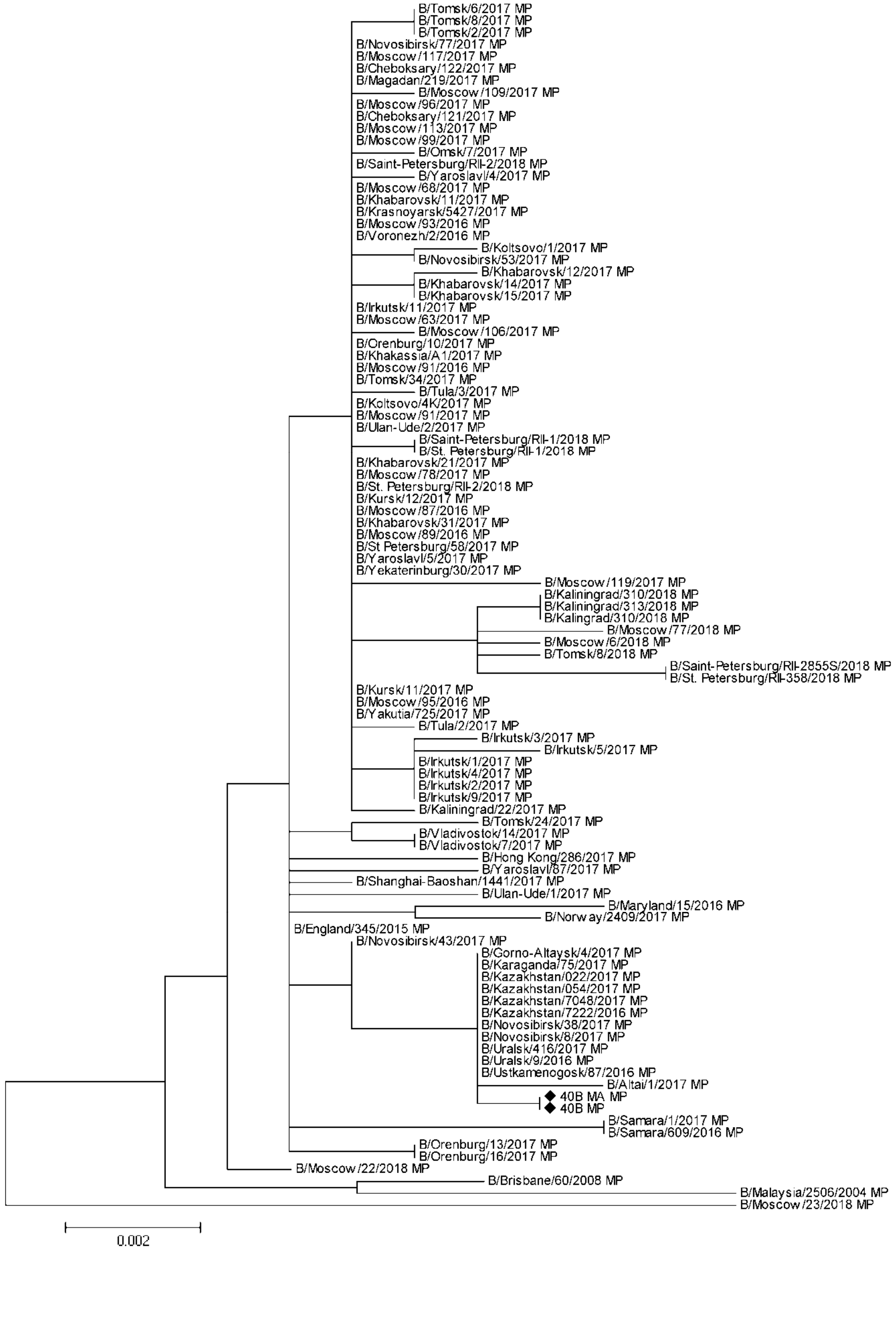

Figure 7. Phylogenetic dendrogram of a genome segment encoding MP of influenza B virus. Studied strains are marked with black diamonds.

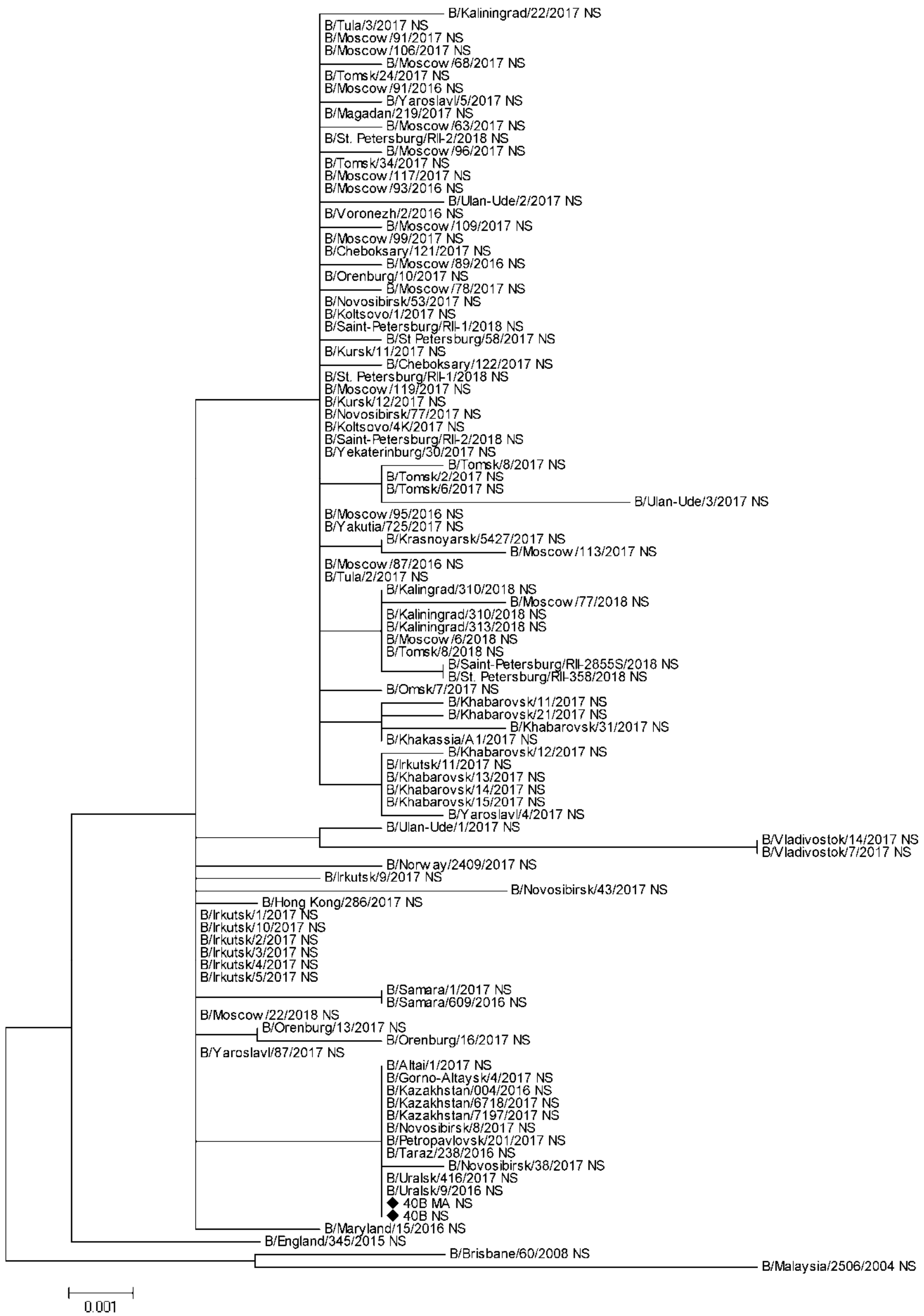

Figure 8. Phylogenetic dendrogram of a genome segment encoding NS of influenza B virus. Studied strains are marked with black diamonds.

Table 1. BLAST-analysis of the influenza B/2017 virus genome segments.

**Segment**	**Name**	**Identity**
HA	B/Altai/1/2017 (B/Vic)	99%
NA	B/Novosibirsk/8/2017 (B/Vic)	100%
PB2	B/Karaganda/75/2017 (B/Vic)	99%
PB1	B/Kazakhstan/022/2017 (B/Vic)	100%
PA	B/Gorna-Altaysk/4/2017 (B/Vic)	99%
NP	B/Kazakhstan/054/2017 (B/Vic)	99%
MP	B/Uralsk/416/2017 (B/Vic)	99%
NS	B/Petropavlovsk/201/2017 (B/Vic)	100%
